# Development and validation of a leukocyte‐associated immunoglobulin‐like receptor‐1 prognostic signature for lower‐grade gliomas

**DOI:** 10.1002/cam4.4945

**Published:** 2022-06-15

**Authors:** Zhansheng Fang, Li Lin, Zewei Tu, Xingen Zhu, Jingying Li, Pengxiang Luo, Kai Huang, Lei Wu

**Affiliations:** ^1^ Department of Neurosurgery The Second Affiliated Hospital of Nanchang University Nanchang Jiangxi People's Republic of China; ^2^ Institute of Neuroscience Nanchang University Nanchang Jiangxi People's Republic of China; ^3^ Department of Comprehensive Intensive Care Unit The Second Affiliated Hospital of Nanchang University Nanchang Jiangxi People's Republic of China

**Keywords:** immune checkpoints, leukocyte‐associated immunoglobulin‐like receptor‐1, overall survival, tumor immune microenvironment

## Abstract

**Objective:**

Leukocyte‐associated immunoglobulin‐like receptor‐1 (LAIR‐1), is an immunosuppressive receptor, widely expressed by immune cells, but the part of LAIR‐1 in glioma progression remains unclear. The purpose of this study was to explore the relationship between LAIR‐1 expression and the development of lower‐grade glioma (LGG) using publicly available data sets.

**Methods:**

We took advantage of The Cancer Genome Atlas (TCGA) to analyze the expression of LAIR‐1 in patients with LGG. Second, Kaplan‐Meier methods and univariate and multivariate Cox regression analyses were used to examine the clinical significance of LAIR‐1 expression in combination with CGGA databases, and then receiver operating characteristic curve analysis was used to verify the prognostic utility of LAIR‐1. Gene ontology (GO), Kyoto Encyclopedia of Genes and Genomes (KEGG) and gene set enrichment analysis (GSEA) were used to explore the function of LAIR‐1. Analysis of the correlation with immune infiltration was conducted using the ESTIMATE algorithm and single sample gene set enrichment analysis.

**Results:**

Our results showed that LAIR‐1 expression to be positively correlated with malignant clinicopathologic features of LGG. Univariate analysis and multivariate analysis revealed that overexpression of LAIR‐1 was correlated with a worse prognosis in patients. A nomogram model combining LAIR‐1 was more useful in guiding clinical diagnosis, and functional enrichment analysis showed that malignant development of glioma was closely affiliated with the tumor immune microenvironment.

**Conclusion:**

These results indicate that LAIR 1 is a latent marker for determining the prognosis of LGG patients. LAIR 1 may also participate a critical part in TIME of LGG by regulating the infiltration of immune cells, suggesting that LAIR 1 might be used as a therapeutic target to regulate the antitumor immune response.

## Graphical abstract

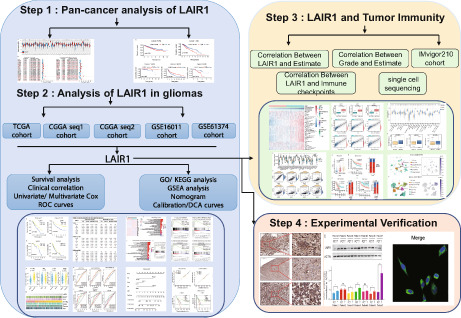

## 1 INTRODUCTION

Glioma is one of the most common primary cancers of the central nervous system, and the incidence of glioma is increasing worldwide. Analysis of the incidence of central nervous system tumors in the United States from 2011 to 2015 indicated that gliomas take the proportion of 26% of all intracranial neoplasms and 81% of intracranial malignancies.^1^ Various biomarkers of glioma have been discovered and characterized in the past few years, with molecular, genetic, and micro‐RNA biomarkers described in glioma patients.^2^ Unfortunately, lower‐grade glioma (LGG) can develop into high‐grade gliomas and become resistant to chemotherapy.^3^ In the past several decades, cancer research has expanded from the study of genetic aspects to broader investigations of the tumor immune microenvironment (TIME). The growth of malignant tumors depends not only on malignant cells, but also chronic inflammation retained by tumor surrounding cells. Tumor‐infiltrating host cells may secrete soluble factors such as cytokines and chemokines that exert pro‐ or anti‐tumor functions.^4^

Leukocyte‐associated immunoglobulin‐like receptor‐1 (LAIR‐1), also known as CD305, is a type I transmembrane glycoprotein of 287 amino acids, its extracellular domain includes a single C2‐type Ig‐like domain, and its intracellular domain embodies two immuno‐receptor tyrosine‐based inhibitory motifs (ITIMs).^5^ LAIR‐1 interacts with multiple functional ligands, such as collagen of extracellular matrix, complement C1q, and surfactant protein D.^6^ It is noteworthy that collagens are functional ligands for LAIR‐1 and directly restrain the activation of immune cells among primary cells.^7^ ITIM inhibitory receptors participate a critical part in modulation of the immune system. LAIR‐1 is an immune inhibitor receptor, and the primary mechanism was through which it acts involves the recruitment of Scr homologous phosphatase‐1 (SHP1) to tyrosine‐based inhibitory motifs following the combining of LAIR‐1 to its ligand, and further enlist of SHP2 and C‐terminal Src kinase leads to transduction of a negative signal.^8^ LAIR‐1 is widely exists on immune system cells including B cells, macrophages, natural killer cells, monocytes, dendritic cells, and CD34^+^ hematopoietic progenitor cells.^9^ In recent years, research has revealed that the LAIR‐1 is not only present in hematopoietic tumors, but also in non‐hematopoietic tumors. In addition, high expression of LAIR‐1 is highly correlated with the malignant degree of the tumor.^10^ To date, however, there have been few studies examining the role of LAIR‐1 in glioma. The correlation between LAIR‐1 and the prognosis of glioma patients was poorly understood in terms of the correlation with the glioma microenvironment, and therefore the specific mechanism needs further study.^11^

The purpose of the present study was to comprehensively examine the mechanism of LAIR‐1 in the progression and prognosis of gliomas and determine its correlation with malignant clinical features. We also explored the effect of LAIR‐1 on the level of immune cell invasion as well as the TIME and evaluated the predictive performance of LAIR‐1 for immune infiltration in glioma.

## 2 MATERIALS AND METHODS

**FIGURE 1:**
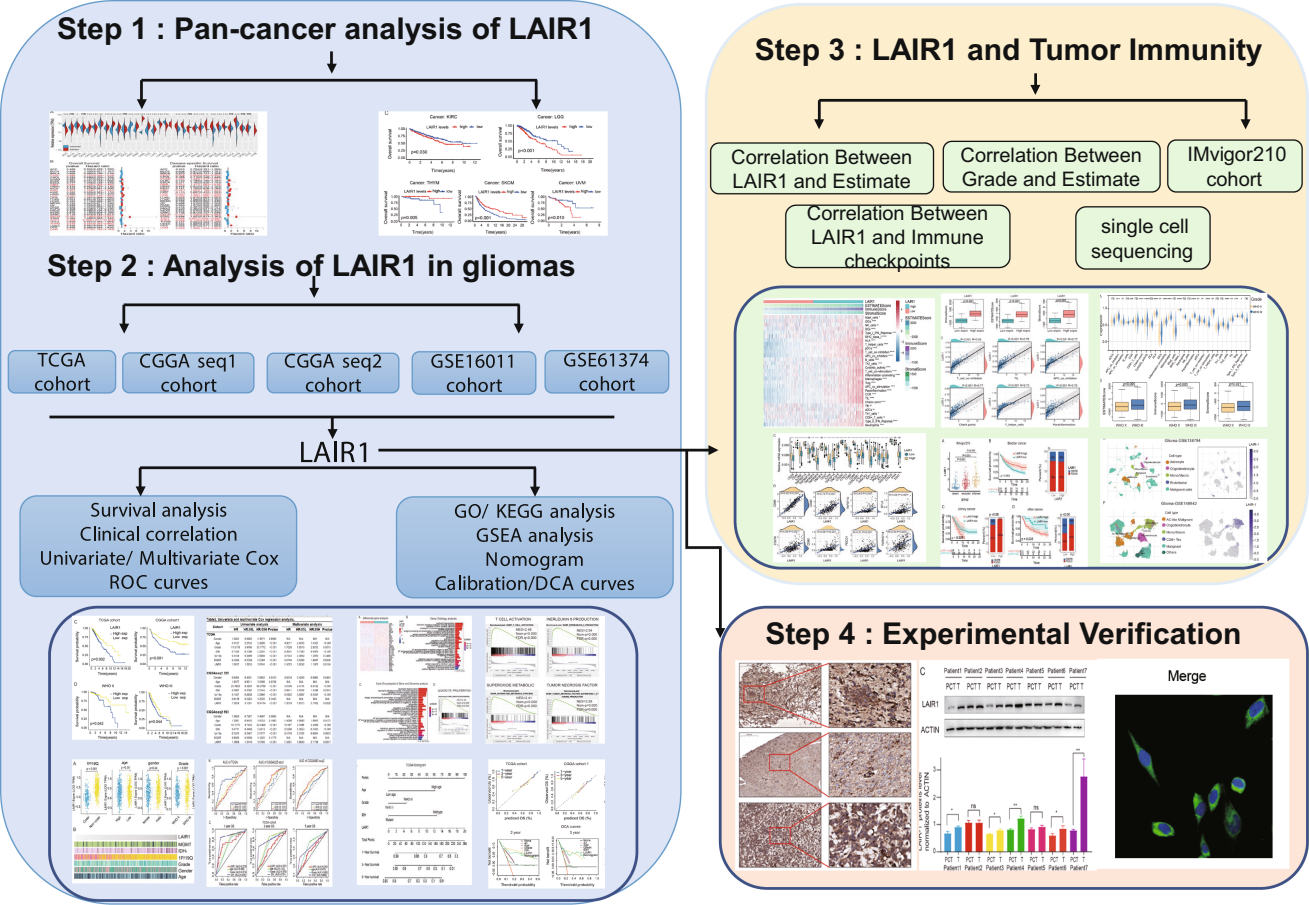
The workflow of this study

The flowchart of this work is shown as Figure 1.

### 2.1 Data acquisition

Five independent glioma cohorts were examined in the present study. For the TCGA dataset, mRNA expression files were acquired from the Genomic Data Commons Data Portal (https://portal.gdc.cancer.gov/), and corresponding clinicopathologic data were gained from the cbioportal website (https://www.cbioportal.org/). As for the CGGA‐325seq1 and CGGA‐693seq2 datasets, RNA‐seq data and associated clinicopathologic data were downloaded from the CGGA website (http://www.cgga.org.cn/). For the GSE16011 and GSE61374 cohorts, RNA‐seq data and associated clinicopathologic data were downloaded from the GEO website (https://www.ncbi.nlm.nih.gov/geo/) and previous publications.^12^, ^13^ Data for the IMvigor210 cohort^14^ were acquired from the website (https://www.nature.com/articles/nature25501). Immunohistochemistry images were downloaded from the Human Protein Atlas databases (https://www.proteinatlas.org/).

All clinical samples of human glioma tissue used in this study were acquired from patients who underwent surgical treatment with subsequent pathological confirmation at the Second Affiliated Hospital of Nanchang University in Jiangxi Province, China. The patients provided their written informed consent to participate in this study.

### 2.2 Patient exclusion criterion

Exclusion criteria for patients with glioma were as follows: (a) Glioma patients lacking overall survival (OS) information or OS <30 days; (b) patients lacking World Health Organization (WHO) classification information or expression data, and (c) patients with WHO grade I glioma or WHO grade IV glioma. According to the exclusion criteria, we examined five RNA‐seq cohorts (TCGA, CGGAseq1, CGGAseq2, GSE16011, and GSE61374 cohorts), which included 422, 420, 171, 103, and 134 glioma patients, respectively. The clinicopathologic and molecular characteristics of the glioma patients contained in the present study are shown in Table 1.

**TABLE 1:** Clinical molecular information of patients with glioma involved in this study

Features	TCGA (*n* = 422)	CGGAseq1 (*n* = 171)	CGGAseq2 (*n* = 420)	GSE16011 (*n* = 103)	GSE61374 (*n* = 134)
Overall survival (years)					
Median (range)	1.58 (0.10–17.60)	5.90 (0.08–13.18)	3.95 (0.14–13.78)	3.32 (0.19–20.68)	4.5 (0.10–17.70)
<5	371 (87.91%)	83 (48.54%)	264 (62.86%)	51 (49.51%)	74 (55.22%)
>=5	51 (12.09%)	88 (51.46%)	156 (37.14%)	52 (50.49%)	60(44.78%)
Age					
Median (range)	41 (14–87)	39 (10–74)	40 (11–72)	43 (23–81)	41 (21–80)
<median age	203(48.10%)	82 (47.95%)	190 (45.24%)	44 (42.72%)	64 (47.76%)
>=median age	219 (51.90%)	89 (52.05%)	229 (54.52%)	58 (56.31%)	70 (52.24%)
NA	0 (0.00%)	0 (0.00%)	1 (0.24%)	1 (0.97%)	0 (0.00%)
Gender					
Male	234 (55.45%)	106 (61.99%)	235 (55.95%)	67 (65.05%)	82 (61.19%)
Female	188 (44.55%)	65 (38.01%)	185 (44.05%)	36 (34.95%)	52 (38.81%)
WHO grade					
WHOII	200 (47.39%)	97 (56.73%)	173 (41.19%)	22 (21.36%)	60 (44.78%)
WHOIII	222 (52.61%)	74 (43.27%)	248 (59.05%)	81 (78.64%)	74 (55.22%)
IDH mutation status					
Mutant	343 (81.28%)	126 (73.68%)	288 (68.57%)	45 (43.69%)	113 (84.33%)
Wild	77(18.25%)	44 (25.73%)	94 (22.38%)	37 (35.92%)	21 (15.67%)
NA	2 (0.42%)	1 (0.58%)	38 (9.05%)	21 (20.39%)	0 (0.00%)
1p/19q codeletion status					
Non‐codeletion	282 (66.82%)	114 (66.67%)	257 (61.19%)	39 (37.86%)	98 (73.13%)
Codeletion	140 (33.18%)	55 (32.16%)	125 (29.76%)	37 (35.92%)	36 (26.86%)
NA	0 (0.00%)	2 (1.17%)	38 (9.05%)	27 (26.21%)	0 (0.00%)
MGMT promoter status					
Methylated	350 (82.94%)	85 (49.71%)	200 (47.62%)	NA	96 (71.64%)
Unmethylated	72 (17.06%)	70 (40.94%)	129 (30.71%)	NA	13 (9.70%)
NA	0 (0.00%)	16 (9.36%)	91 (21.67%)	NA	25(18.66%)

### 2.3 Analysis of correlations between LAIR‐1 expression and clinical characteristics

To verify the prognostic value of LAIR‐1 in LGG, five independent datasets from the TCGA, CGGA, and GEO databases were downloaded. In the TCGA cohort, 422 LGG patients (mean age 41 years, range:14–87 years) were included. The CGGAseq1 and CGGAseq2 cohorts were used to validate the prognostic value of LAIR‐1, which included 420 and 171 patients (mean age 40 and 39 years, respectively; range: 11–72 and 10–74 years, respectively). The relationships between LAIR‐1 expression and clinical outcomes of patients were determined via univariate and multivariate Cox regression analyses (Table 2). Kaplan–Meier survival analysis was utilized to contrast the OS of patients with high and low LAIR‐1 expression in the five datasets (TCGA dataset, the CGGA, and the GEO databases). The predictive power of LAIR‐1 expression in terms of prognosis was estimated by receiver operating characteristic (ROC) curves. The R packages “rms” and “foreign” were used to formulate the nomograms, and then the performance of the nomograms was evaluated using the calibration curves. Decision curve analysis (DCA) was implemented to assess the prediction efficiency of the nomograms.

**TABLE 2:** Univariate and multivariate Cox regression analysis

Cohort	Univariate analysis				Mutivariate analysis			
	HR	HR.95 L	HR.95H	*p* value	HR	HR.95 L	HR.95H	*p* value
TCGA								
Gender	1.0224	0.6983	1.4971	0.9093	N/A	N/A	N/A	N/A
Age	3.4137	2.2155	5.2600	<0.001	4.2617	2.4435	7.4327	<0.001
Grade	11.9178	3.9706	35.7712	<0.001	1.7528	1.0518	2.9212	0.0313
IDH	0.1506	0.1006	0.2255	<0.001	0.2601	0.1360	0.4975	<0.001
1p/19q	0.4100	0.2469	0.6808	<0.001	0.7343	0.3859	1.3970	0.3466
MGMT	0.3566	0.2358	0.5394	<0.001	0.9749	0.5266	1.8047	0.9350
LAIR1	1.5917	1.2833	1.9741	<0.001	1.1073	1.0242	1.1971	0.0104
CGGAseq1 325								
Gender	0.6494	0.4281	0.9850	0.0423	0.6518	0.4256	0.9983	0.0491
Age	1.8377	0.9511	3.5508	0.0702	N/A	N/A	N/A	N/A
Grade	23.4920	8.3265	66.2788	<0.001	3.8109	2.4131	6.0182	<0.001
IDH	0.3697	0.2382	0.5740	<0.001	0.8911	0.5557	1.4288	0.6321
1p/19q	0.1567	0.0828	0.2966	<0.001	0.1822	0.0927	0.3581	<0.001
MGMT	0.8119	0.5263	1.2526	0.3463	N/A	N/A	N/A	N/A
LAIR1	7.5659	3.1091	18.4114	<0.001	1.8318	1.0515	3.1912	0.0326
CGGAseq2 693								
Gender	1.0600	0.7987	1.4067	0.6866	N/A	N/A	N/A	N/A
Age	1.2081	0.9103	1.6033	0.1905	1.4590	1.0690	1.9915	0.0173
Grade	10.3175	4.7433	22.4426	<0.001	3.1187	2.1691	4.4839	<0.001
IDH	0.4711	0.3458	0.6418	<0.001	0.5200	0.3653	0.7403	<0.001
1p/19q	0.3573	0.2467	0.5177	<0.001	0.5170	0.3331	0.8024	0.0033
MGMT	0.8065	0.5900	1.1025	0.1776	N/A	N/A	N/A	N/A
LAIR1	1.3959	1.2410	1.5703	<0.001	1.5201	1.0630	2.1738	0.0217
GSE16011								
Gender	0.9329	0.5951	1.4624	0.7619	N/A	N/A	N/A	N/A
Age	1.0348	1.0166	1.0533	<0.001	1.0422	1.0208	1.0641	0.0001
Grade	1.0813	0.6153	1.9000	0.7858	N/A	N/A	N/A	N/A
IDH1	0.8214	0.5034	1.3401	0.4307	N/A	N/A	N/A	N/A
1p19q	0.4560	0.2744	0.7578	0.0024	0.5145	0.2733	0.9689	0.0396
LAIR1	0.5803	0.3757	0.8963	0.0141	0.6507	0.3310	1.2793	0.2129
GSE61374								
Gender	1.2220	0.6509	2.2941	0.5327	N/A	N/A	N/A	N/A
Age	1.0555	1.0313	1.0802	<0.001	1.0704	1.0431	1.09837	<0.001
Grade	1.5103	0.8055	2.8319	0.1986	N/A	N/A	N/A	N/A
IDH1/2	0.2615	0.1364	0.5012	<0.001	0.4875	0.2449	0.970084	0.04071
1p/19q	0.2389	0.0851	0.6702	0.0065	0.2419	0.0801	0.730764	0.01187
MGMT	0.5382	0.2737	1.0584	0.0726	N/A	N/A	N/A	N/A
LAIR1	0.4876	0.2627	0.9049	0.0228	0.4905	0.2531	0.950575	0.03485

### 2.4 Function enrichment analysis

We attempted to identify the signaling pathways by functional enrichment analysis. The R‐package “LIMMA” was used to sort differentially expressed genes (DEGs) through the TCGA database. The screened DEGs were examined using the R packages “cluster Profiler”, “Rich Plot” and “ggplot2” for Gene Ontology (GO), Kyoto Encyclopedia of Genes and Genomes (KEGG) pathway analysis, and gene set enrichment analysis (GSEA).^15^

### 2.5 ESTIMATE algorithm and single sample gene set enrichment analysis

The ESTIMATE algorithm, which assessed stromal and immune cells in pernicious tumor tissues using expression data, was used to acquire immune‐related scores to predict the infiltration of immune cells in LGG.^16^ The analytical method is contained in the “estimated” R package. we also quantify the enrichment of 29 immune‐related features by using single sample GSEA, and identified the related effects of LAIR‐1 expression on infiltrate of immune cells in the TIME of LGGs.

### 2.6 Single‐cell RNA sequencing analysis

The distribution and abundance of LAIR‐1 in various cell types: Astrocyte, oligodendrocyte, mono/macro cell, endothelial cell, CD8^+^ cell, and malignant cell, was performed by Single‐cell RNA sequencing analysis. The single‐cell RNA sequencing analysis of the GSE138794 cohort and GSE148842 cohort were performed in the Tumor Immune Single‐cell Hub (TISCH) website (http://tisch.comp‐genomics.org/).

### 2.7 Western blot analysis and quantitative real‐time PCR (qPCR)

We extracted total protein from radioimmunoprecipitation assay buffer (RIPA) with phosphatase inhibitor (Solarbio, P1260) and phenylmethanesulfonylfluoride (PMSF) (Solarbio, P0100), then electrophoresed at 90 V for 30 min and 120 V for 1 h. Proteins were transferred onto a polyvinylidene difluoride membrane (PVDF) membrane, (Millipore, IPVH00010) which was soaked in methanol and blocked for 2 h. The PVDF membrane was incubated with antibodies against LAIR‐1 (1:4000, 67200‐1‐Ig, Proteintech) and GAPDH (1:3000, 10494‐1‐AP, Proteintech) for 14 h and then incubated with anti‐rabbit and anti‐mouse secondary antibodies. For qPCR analysis, total RNA was extracted using an extraction kit (simply, BSC5M1). And then extracted RNA was reverse transcribed to cDNA with Prime Script RTase (Ribobio). FastStart Universal SYBR Green Master (Roche Diagnostics) was used to implement qRT‐PCR. The primers were as follows: Forward primer for LAIR‐1 5′‐GCCAGAATCAGATAAAGCAGG‐3′; and reverse primer for LAIR‐1 5′‐CTGAGCATACGTCACCTCCT‐3′; forward primer for GAPDH 5′‐CAGGAGGCATTGCTGATGAT‐3′; and reverse primer for GAPDH 5′‐GAAGGCTGGGGCTCATTT‐3′.

### 2.8 Cell culture

Bt142 mut/−, SW‐1783, and SW‐1088 human glioma cell lines were obtained from the American Type Culture Collection (ATCC). Normal human astrocytes (NHA) were received from the Culture Collection of the Chinese Academy of Sciences (Shanghai, China). Leibovitz's L‐15 medium (ATCC) supplemented with 10% fetal bovine serum (GIBCO, Thermo Fisher Scientific) was applied to culture SW‐1783 and SW‐1088 cells. Bt142 mut and NHA cell lines were maintained in Dulbecco's modified Eagle medium/F12 medium. NHA cells were cultured in an incubator with 5% CO2 at 37°C, whereas Bt142 mut/−, SW‐1783, and SW‐1088 human glioma cells were cultured in an incubator under normal oxygen conditions at 37°C.

### 2.9 Immunofluorescence assay

First, SW‐1088 cells were allowed to climb cell climbing sheets in a 6 well plate, fixed at room temperature with 4% paraformaldehyde (Solarbio, P1110) for 15 min, washed with PBS three times, permeabilized with 0.5% triton x‐100 (Solarbio, T8200) for 20 min at room temperature. Next, the sheets were blocked with 10% goat serum for 1 h and washed three times with PBS. The cells were then incubated with the rabbit anti‐LAIR‐1 (LAIR‐1, 1: 400, 67220‐1‐Ig, Proteintech) at 4°C overnight and then with fluorescent anti‐rabbit IgG (H + L) antibody (1:200, Jackson ImmunoResearch) in the dark at room temperature for 1 h. Finally, nuclei were counterstained with DAPI for 40s followed by phalloidin staining for 10 min. The cells were photographed using confocal laser scanning microscopy (Nikon, C2si/C2).

### 2.10 Immunohistochemistry assay

Glioma tissues and adjacent normal tissues were made into paraffin sections, dewaxed with xylene, and hydrated with 95% ethanol. Then, the sections were repaired by immersion of endogenous peroxidase at 37.0°C in citric acid buffer for 30 min, blocked with 5% goat serum at room temperature for 60 minutes, and incubated with antibodies against LAIR‐1 (1:4000, 67200‐1‐Ig, Proteintech). Next, the horseradish peroxidase (HRP)‐conjugated secondary antibody was applied at 37°C for 45 minutes and diaminobenzidine (DAB) solution (Beyotime) was used to stain the slice, and we counterstained the nuclei with hematoxylin (Beyotime). Immunohistochemical staining was independently evaluated by two pathologists without prior knowledge of patient characteristics. According to the degree of positive staining (antigen content), it can be divided into: Weak positive (+); Moderate positive (++); Strong positive (+++). According to the number of positive cells, they can be divided into: Weakly positive (+, <25%); Medium positive (++, 25%–49%); Strong positive (+++, >50%).

### 2.11 Statistical analysis

Based on the TCGA and CGGA datasets, Kaplan–Meier curve analysis was performed to compare the clinical outcomes between low‐expression LAIR‐1 subgroup and high‐expression LAIR‐1 subgroup and examine the correlations between various clinicopathologic features and OS. OS was evaluated by using time‐dependent ROC curves. Additionally, univariate, and multivariate Cox regression analyses were conducted to assess the factors associated with prognosis in LGG patients. The analyses were performed using SPSS software 26.0 and R software of RStudio (https://www.rstudio.com/). Differences were considered significant at *p* < 0.05.

## 3 RESULTS

### 3.1 Potential prognostic significance of LAIR‐1 expression in different human cancers

**FIGURE 2:**
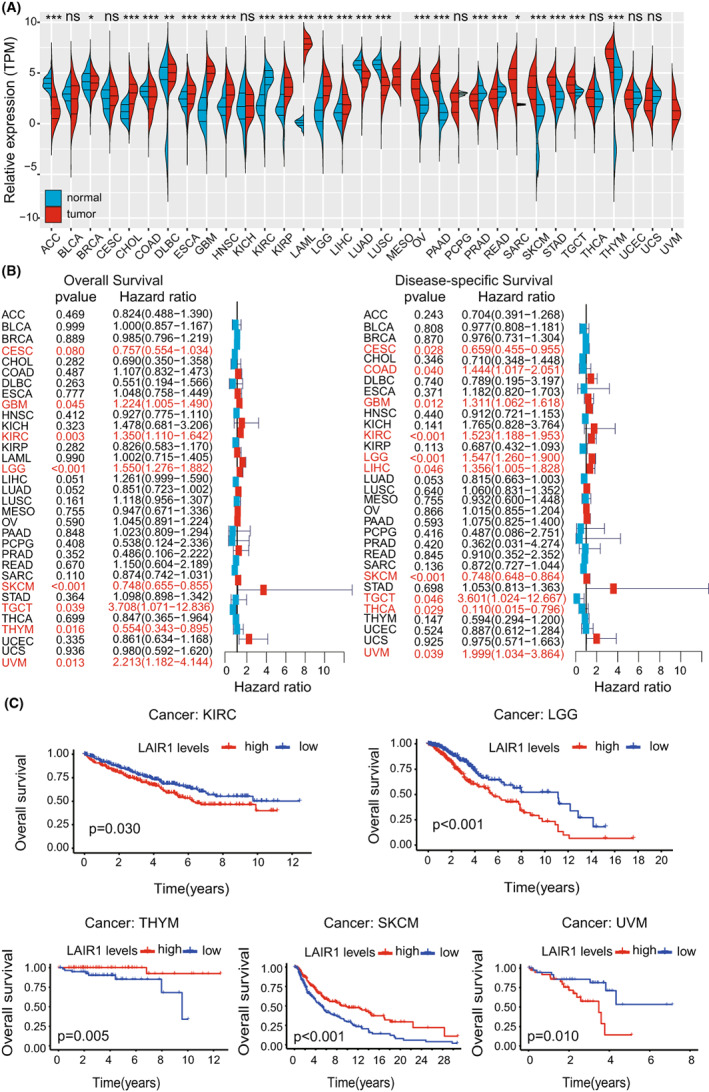
(A) Difference analysis of LAIR‐1 expression in 33 different types of tumors. (*p < 0.05, **p < 0.01, and ***p < 0.001) (B) Prognostic significance of LAIR‐1 in different malignant tumors. (C) High expression of LAIR‐1 in five diverse tumors were associated with poor OS based on the TCGA dataset.

We initially found that LAIR‐1 was differentially expressed in many tumors (Figure 2A). To explore the prognostic significance of LAIR‐1 expression in different malignant tumors, univariate Cox regression analysis was performed on LAIR‐1 mRNA expression in 33 malignant tumors in the TCGA dataset. The analysis revealed that LAIR‐1 was a risk factor for LGG, glioblastoma (GBM), kidney renal clear cell carcinoma (KIRC), thymoma (THYM), skin Cutaneous Melanoma (SKCM), and uveal Melanoma (UVM) (Figure 2B). Further, the survival analysis indicated that high LAIR‐1 expression was interrelated with poor OS of patients with KIRC, LGG, and UVM. However, low LAIR‐1 expression was interrelated with poor OS of patients with THYM and SKCM (Figure 2C).

### 3.2 The Prognostic Value of LAIR‐1 Expression in LGG Patients

**FIGURE 3:**
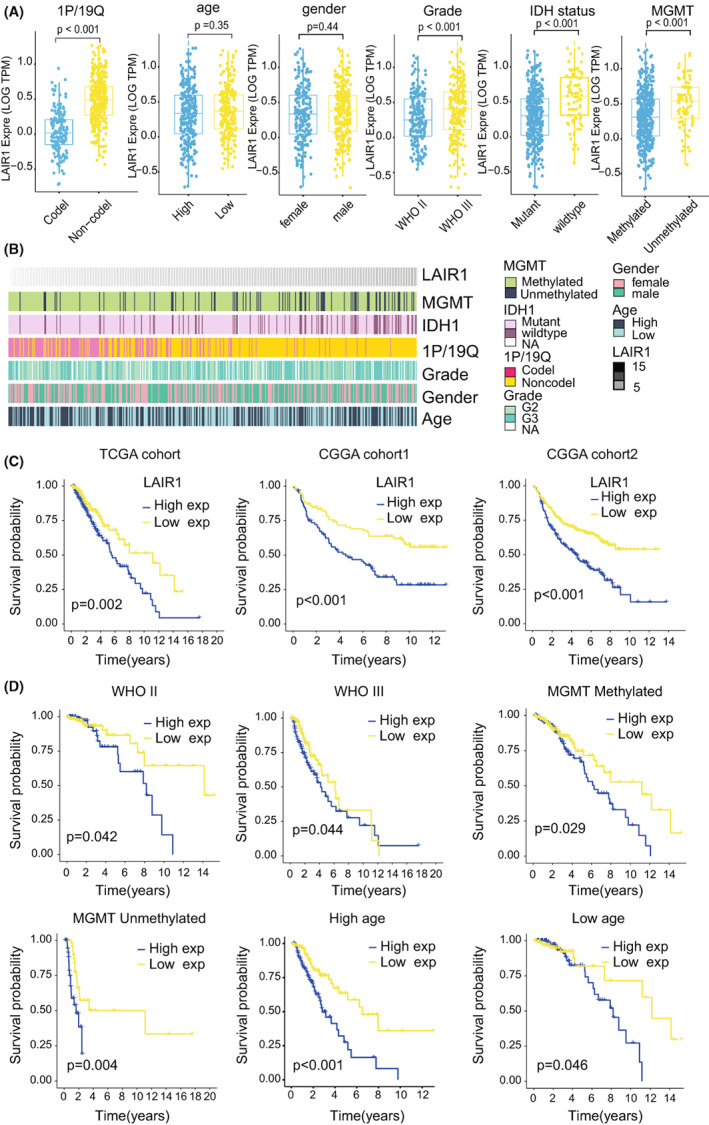
(A) Based on TCGA database, LAIR‐1 expression differed among various subgroups, except for gender and age. (B) Heatmap showing the dependence between the expression level of the LAIR‐1 and clinicopathologic characteristics containing 1p/19q status, age, gender, grade, IDH status, and MGMT. (C) Kaplan–Meier survival analysis was utilized to contrast the OS of patients with high LAIR‐1 expression and low LAIR‐1 expression in the TCGA and CGGA datasets. (D) Relationship between LAIR‐1 expression and clinical characteristics: Age, MGMT status, and grade in the TCGA dataset.

In the above, we reported that LAIR‐1 may have prognostic significance in LGGs. In order to further explore potential malignant behavior of LAIR‐1, a total of 1013 patients with LGG in the TCGA and CGGA databases were analyzed. First, we compared the differential level of LAIR‐1 expression in different subgroups stratified by 1p/19q status, age, gender, grade, IDH status, and O‐6‐methylguanine DNA methyltransferase (MGMT). As shown in Figure 3A, based on the TCGA database, LAIR‐1 expression was significantly different among subgroups stratified by 1p/19q status (*p* < 0.0001), grade (*p* < 0.001), IDH status (*p* < 0.0001), and MGMT (*p* < 0.0001), but not in subgroups stratified by age (*p* = 0.35) or gender (*p* = 0.44). In the heatmap for the relationship between LAIR‐1 expression level and clinicopathologic characteristics, the expression of LAIR‐1 was positively interrelated with unmethylated and wild‐type IDH, whereas chromosome code 1p/19Q was negatively correlated (Figure 3B). In the two validation databases CGGAseq1 and CGGAseq2, LAIR‐1 expression was similar to the TCGA database, except for the MGMT subgroups (Figures S1A and S2A).

Univariate and multivariate analyses showed that LAIR‐1 expression was a separate prognostic indicator in LGG patients (TCGA Cohort: HR = 1.1073, 95% CI = 1.0242–1.1971, *p* = 0.0104, CGGAseq1 Cohort: HR = 1.8318, 95% CI = 1.0515–3.1912, *p* = 0.0326, CGGAseq2 Cohort: HR = 1.5201 95% CI = 1.0630–2.1738, *p* = 0.0217, Table 2). Kaplan–Meier survival analysis was conducted to contrast the OS of patients with high and low expression of LAIR‐1 and determine whether LAIR‐1 can be used as a prognostic marker for LGG patients. The similar results of the TCGA, CGGA, and GEO databases showed that patients with high LAIR‐1 expression had poorer OS (Figure 3C; Figure S4A,E). In addition, stratified survival analysis was performed according to WHO grade, MGMT status, and age to assess the prognostic importance of LAIR‐1 in different subgroups of glioma patients. The results of stratified survival analyses were consistent with the OS in the three independent databases (Figure 3D; Figures S1B, S2B, and S3A). The univariate ROC curves were utilized to estimate the model fit in the TCGA, CGGA and GEO cohorts. The area under curve (AUC) of univariate ROC for 1‐, 3‐, and 5‐years OS were 0.724, 0.585, 0.540, respectively (Figure S3B). It also showed a prominent fitting prediction in CGGA and GEO datasets (Figures S3B, S4B and S4F). Otherwise, the area under curve (AUC) of multivariate ROC for 1‐years OS regarding to LAIR‐1, Age, Grade, and IDH were 0.725, 0.699, 0.674, and 0.854 in the TCGA cohort, respectively (Figure S3C). The multivariate ROC for 1‐, 3‐, and 5‐years OS in the CGGA cohorts were shown in Figure S3D. The multivariate ROC for 1‐, 3‐, and 5‐years OS in the GEO cohorts were shown in Figures S4C and S4G.

### 3.3 Function enrichment analysis

**FIGURE 4:**
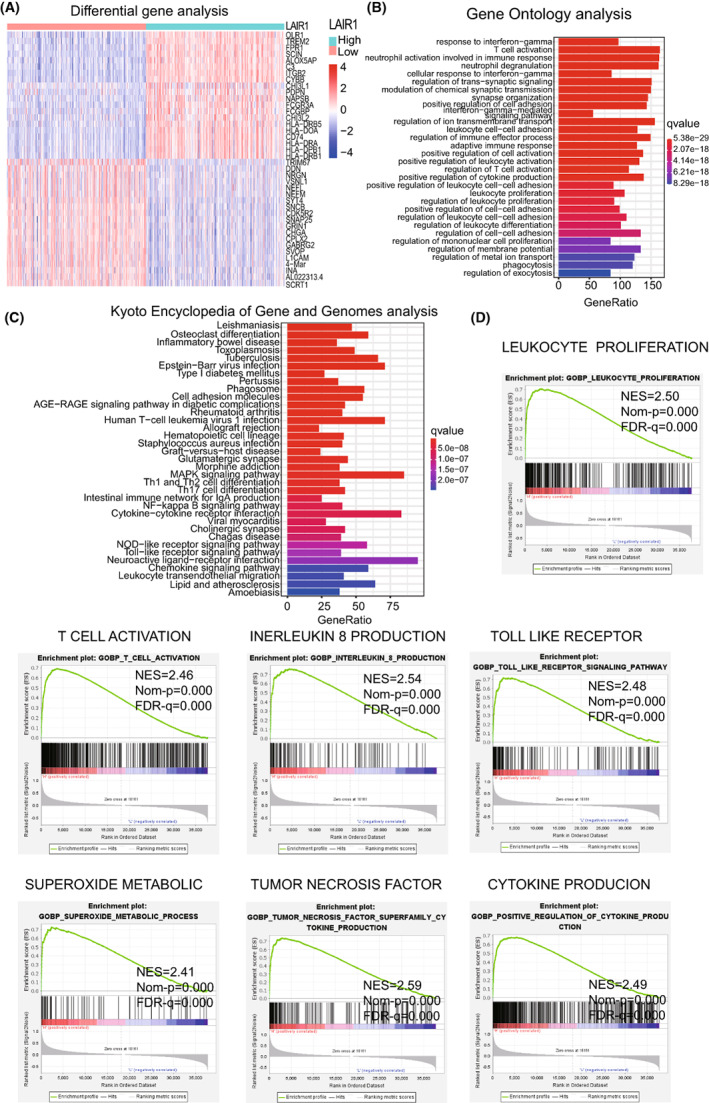
(A) Functional enrichment analysis of DEGs between the low and high LAIR‐1 expression groups. (B) GO analysis and (C) KEGG analysis. (D) Immune‐response characteristics of associated with highly expression LAIR‐1 were identified by GSEA.

A differential expression analysis was conducted for the low and high LAIR‐1 expression subgroups in the TCGA cohort to investigate the potential biological role of LAIR‐1. A total of 3439 DEGs were recognized with standard of | log2 (fold change) | >2 and *p*‐value <0.05 (Figure 4A). These DEGs were subjected to GO and KEGG pathway analyses taking advantage of the R package “cluster Profiler, enrich plot, ggplot2”. The DEGs were chiefly enriched in immunocyte‐related bioprocesses such as adaptive immune response, T cell activation, and regulation of immune effector process (Figure 4B). Additionally, the DEGs were also chiefly enriched in leukocyte trans‐endothelial migration, neuroactive ligand−receptor interaction, and cytokine−cytokine receptor interaction pathway (Figure 4C). GSEA was conducted to determine whether there was a significant relationship between biological characteristics and the expression level of LAIR‐1, The results suggested that the leukocyte proliferation pathway (Normalize enrichment score (NES) = 2.50, normalize *p* value (Nom‐*p*) = 0.000, adjusted *p* value (FDR‐*q*) = 0.000), T‐cell activation pathway (NES = 2.46, Nom‐*p* = 0.000, FDR‐*q* = 0.000), interleukin (IL)‐8 production pathway (NES = 2.54, Nom‐*p* = 0.000, FDR‐*q* = 0.000), toll‐like receptor pathway (NES = 2.48, Nom‐*p* = 0.000, FDR‐*q* = 0.000), superoxide metabolic pathway (NES = 2.41, Nom‐*p* = 0.000, FDR‐*q* = 0.000), tumor necrosis factor pathway (NES = 2.59, Nom‐*p* = 0.000, FDR‐*q* = 0.000), and cytokine production pathway (NES = 2.49, Nom‐*p* = 0.000, FDR‐*q* = 0.000) were differentially enriched in high‐expression LAIR‐1 phenotypes (Figure 4D). The function enrichment analysis results strongly indicated that LAIR‐1 is closely related to the TIME, and thus worthy of further analysis.

### 3.4 Establishment and validation of a nomogram model for predicting clinical outcomes

**FIGURE 5:**
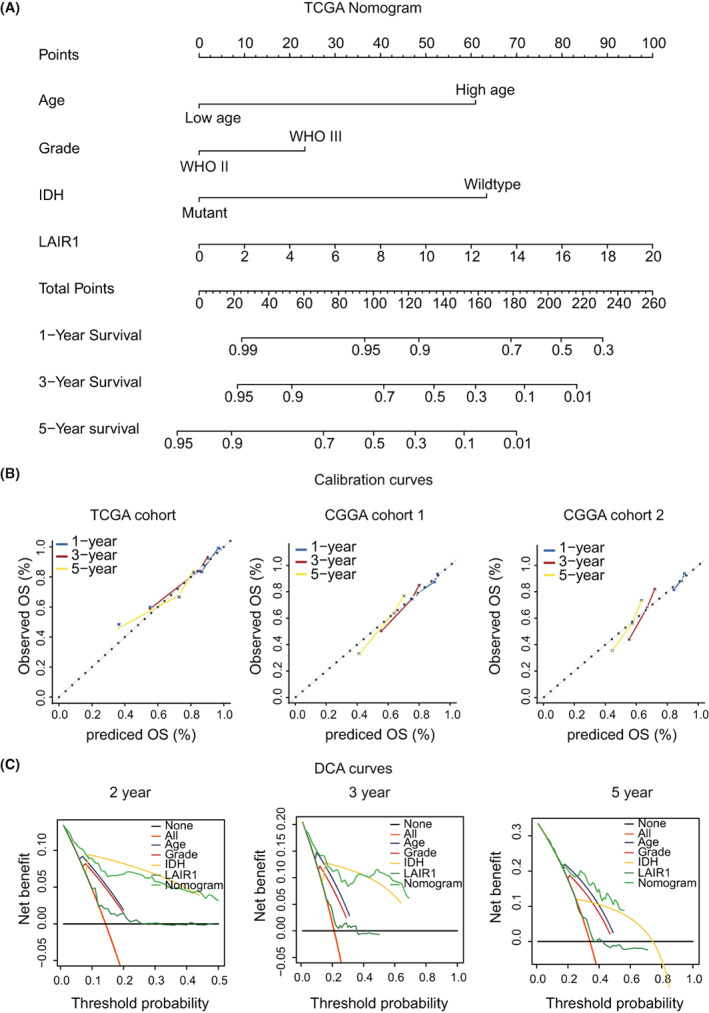
(A) Nomogram constructed to predict patient outcomes. (B) Calibration curves for validation of the nomogram for forecasting patient survival at 1 year, 3 years, and 5 years. (C) Based on TCGA data, DCA curves for prognosis evaluation at 2, 3, and 5 years were constructed based on nomogram, LAIR‐1, IDH, grade, and age.

We constructed a nomogram, a quantitative model for predicting clinical prognosis, to predict 1‐year, 3‐year, and 5‐year OS in the LGG patients of the TCGA dataset using four prognostic factors including age, grade, IDH status, and LAIR‐1 expression (Figure 5A). The calibration curves indicated that the nomogram accurately predicted the 1‐, 3‐, and 5‐year OS of glioma patients in CGGA cohort seq1 and CGGA cohort seq2 (Figure 5B). The similar results are verified in GEO dataset (Figures S4D and S4H). DCA serves as another type of predictive model that is commonly used to evaluate the accuracy of nomograms.^17^ The DCA demonstrated that the net benefit of the nomogram was greater for predicting 2‐year, 3‐year, and 5‐year OS (Figure 5C). Therefore, the multi‐factorial nomogram model including LAIR‐1 exhibited excellent ability to forecast survival in clinical management of LGG patients.

### 3.5 Correlation analysis of immune cell infiltration in TIME of LGG

**FIGURE 6:**
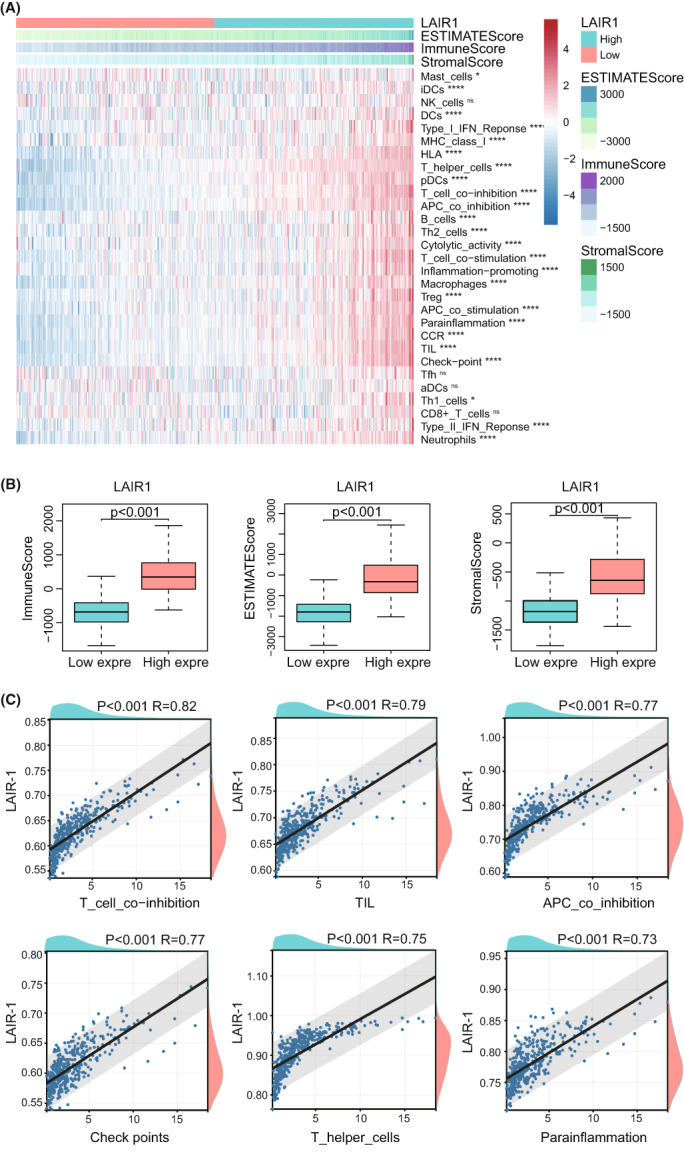
(A) Heatmap demonstrating that the dependence between LAIR‐1 expression level and ESTIMATE‐score, Stromal‐score, and Immune‐score in the TIME, as well as the degree of infiltration of 29 types of immune cells. *p < 0.05, **p < 0.01, ***p < 0.001, ****p < 0.0001. ns (non‐sense) (B) Box plots indicating significant differences in LAIR‐1 expression between ESTIMATE score, Stromal score, and Immune score. (C) Correlation analysis of LAIR‐1 expression among TIL, T cell co‐inhibition, APCs co‐inhibition, check points, T helper cells, and para‐inflammation.

**FIGURE 7:**
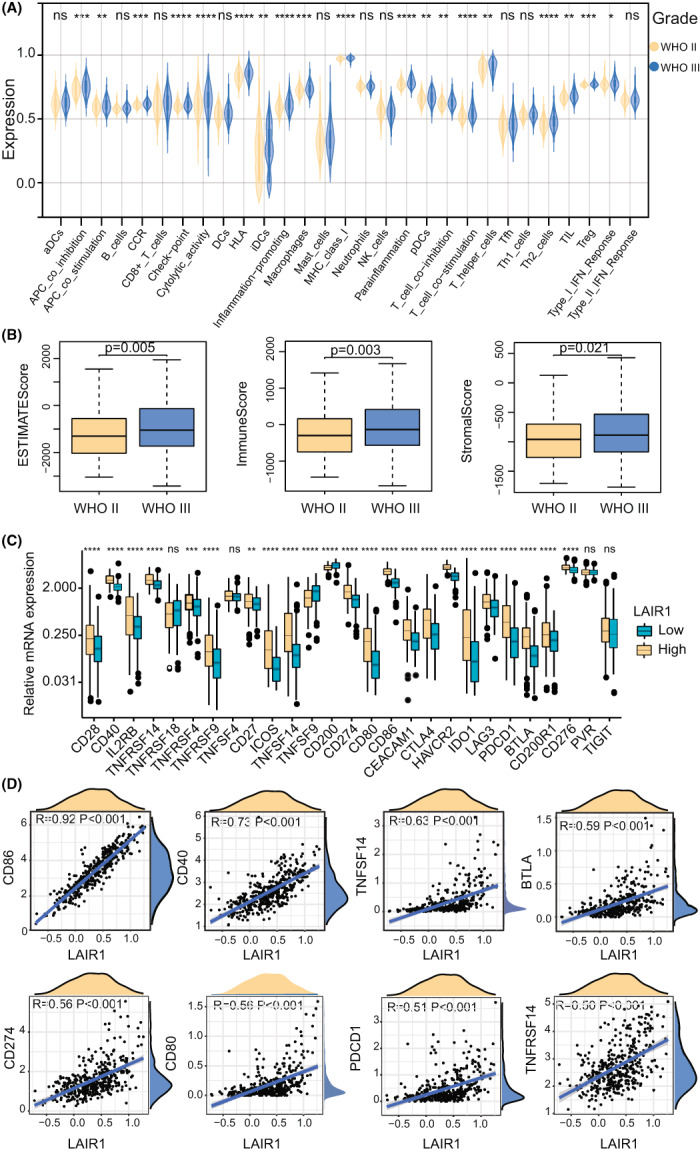
(A) Heatmap demonstrating that the relationship between grade of LGG and infiltration of 29 types of immune cells. *p < 0.05, **p < 0.01, ***p < 0.001, ****p < 0.0001. ns (not significant). (B) Box plots indicating significant differences in grade of LGG between ESTIMATE score, Stromal score, and Immune score. (C) Difference in expression levels of 27 immune checkpoint genes in LGG with high and low LAIR‐1 expression. (D) Expression of LAIR‐1 was significantly positively associated with expression levels of the *CD86, CD40, TNFSF14, BTLA, CD274, CD80, PDCD1, and TNFRSF14* gene in LGG.

Using the ESTIMATE algorithm, the distribution of immunocytes in patients with LGGs was plotted in a bar chart, which manifesting that the distribution of many immune cell types varied significantly in the low and high LAIR‐1 expression subgroups (Figure 6A). In addition, it indicated that the relative abundance of most of the infiltrating immune cell types increased with LAIR1 expression as well as immune‐related scores in the TCGA datasets (Figure 6B). The effects of LGG grade and LAIR‐1 expression on immune infiltration were then analyzed. We discovered that scores (ESTIMATE, stromal, and Immune) were notably higher with Grade III glioma than with Grade II glioma (Figure 7B). Meanwhile, the immune infiltration with Grade III glioma was also abundant than that with Grade II glioma (Figure 7A). It revealed that LAIR‐1 expression level and LGG grade of patients are interrelated to the tumor immunity response. In addition, correlation analysis indicated that the immune gene sets, such as tumor‐infiltrating lymphocyte (TIL), T cell co‐inhibition, antigen‐presenting cells (APCs) co‐inhibition, check points, T helper cells, and para‐inflammation, were significantly correlated with LAIR‐1 expression, and the R coefficients were greater than 0.7 (Figure 6C). The correlation of other immune cells was shown in Figure S5A. These results suggested that the infiltration of immune cell subtypes is closely interrelated with the prognosis of LGG.

### 3.6 Correlation analysis between immune checkpoints and LAIR‐1 expression

Based on patients with LGG in the TCGA cohort, the relationship between LAIR‐1 expression and immune checkpoint genes was investigated. We found that LAIR‐1 expression was positively interrelated with immune checkpoint genes, such as cluster of Differentiation 86 (CD86), cluster of Differentiation 80 (CD80), and tumor necrosis factor (TNF) receptor superfamily member 14 (TNFRSF14) (Figure 7C). As shown in Figures 7D and S6A, LAIR‐1 expression was strongly correlated with CD86, moderately with TNF Superfamily Member 14 (TNFSF14), B‐ and T‐lymphocyte attenuator (BTLA), programmed cell death 1 ligand 1 (PD‐L1; also known as CD274), CD80, programmed cell death 1(PDCD1), and TNFRSF14. These results further validated the hypothesis that LAIR‐1 expression may regulates on the immune response to gliomas.

### 3.7 Prognostic validation of LARI‐1 expression in the immune IMvigor210 cohort and single‐cell RNA sequencing analysis

**FIGURE 8:**
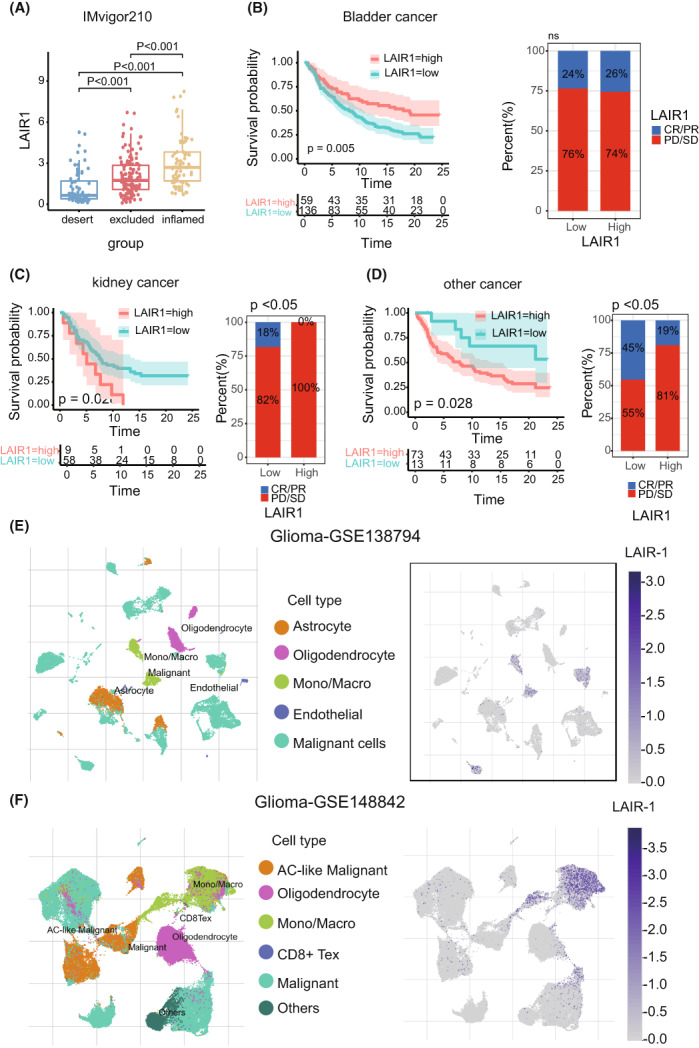
(A) Variance analysis of LAIR1 expression in inflamed, excluded, and desert immune subtypes based on the immune IMvigor210 cohort. (B–D) The survival and statistical analysis of patients with LGGs in different cancer: Bladder cancer, kidney cancer, and other cancer. (E–F) Single‐cell RNA sequencing analysis of LAIR‐1 expression.

Various histologically and transcriptionally immune tumor subtypes were distinguished, including inflamed, excluded, and desert immune tumors.^18^ LAIR‐1 expression also varied statistically in the three immune tumor subtypes (Figure 8A), suggesting that LAIR‐1 expression was inextricably linked to the proposed immune subtypes. Based on the IMvigor210 cohort, we performed survival analyses according to different cancer types. Patients with high LAIR‐1 expression had a worse clinical prognosis for kidney cancer and other cancers (Figure 8B–D). In the kidney cancer group, the proportions of complete response (CR)/partial response (PR), and stable disease (SD)/progressive disease (PD) were 18.00 and 82.00% in the low LAIR‐1 expression group and 0.00 and 100.00% in the high LAIR‐1 expression group, correspondingly (*p* < 0.05) (Figure 8C). Although the immune IMvigor210 cohort was a BLCA immunotherapy cohort (*n* = 398), the predictive effect of LAIR‐1 expression in BLCA can still be explored to elucidate its significance in glioma. Because no immunotherapy for glioma is yet available, understanding the immunology of glioma is of critical importance. Using the single‐cell RNA sequencing analysis, we discovered that LAIR‐1 is highly expressed in macrophages or monocyte compared with other cell types, such as oligodendrocyte, malignant cells, and astrocyte (Figure 8E–F).

### 3.8 In vivo experimental validation

**FIGURE 9:**
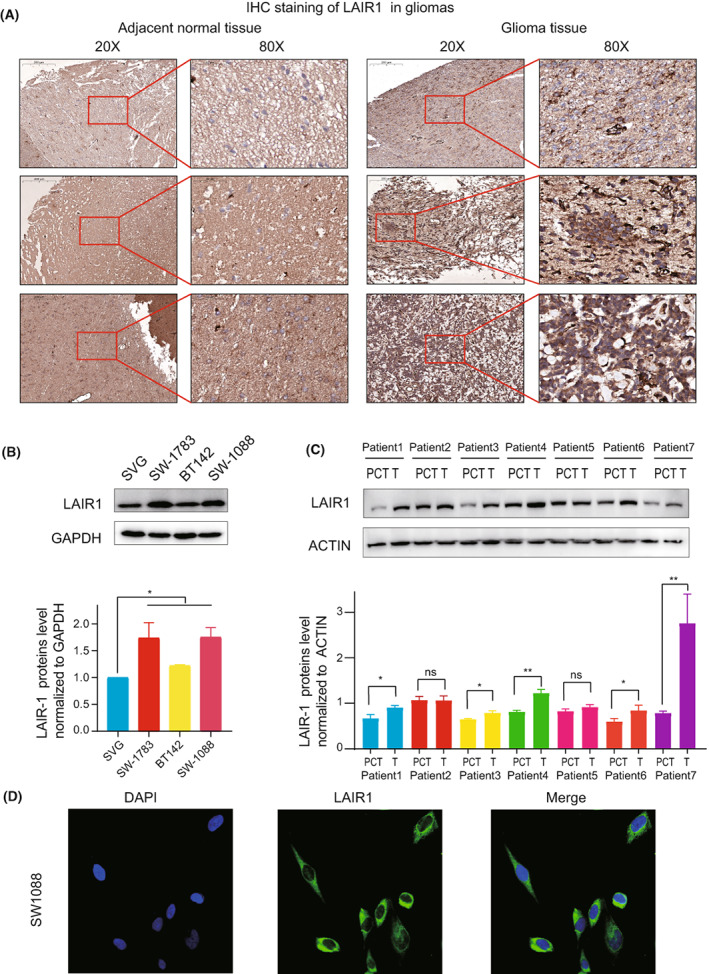
(A) Representative images of IHC staining for LAIR‐1 protein on gliomas tissues and normal tissues. (B) LAIR‐1 protein expression in glioma cells and normal glia cells. The LAIR‐1 protein expression levels were quantified by ImageJ software. Unpaired t‐test was used to analyze. *p < 0.05, **p < 0.01, ***p < 0.001, ****p < 0.0001. ns (non‐sense) (C) LAIR‐1 protein expression in patients with LGG and the matched adjacent tissues. The LAIR‐1 protein expression levels were quantified by ImageJ software. Unpaired t‐test was used to analyze. *p < 0.05, **p < 0.01, ***p < 0.001, ****p < 0.0001. ns (non‐sense) (D) Immunofluorescence images of LAIR‐1 expression in SW‐1088 cell.

Analyses of immunohistochemical images of gliomas indicated that the protein level of LAIR‐1 in glioma tissues was significantly higher than those in adjacent normal tissues (Figure 9A; Figure S6B). Meanwhile, we also confirmed that LAIR‐1 protein was overexpressed in glioma cell lines compared with normal glial cells (NHA) (Figure 9B). Similarly, we took seven LGG tissues and para‐cancerous tissues of same patients in the Second Affiliated Hospital of Nanchang University and found LAIR‐1 protein was highly expressed in the tissues of LGG (Figure 9C). The LAIR‐1 protein was quantified by Image J. Unpaired *t*‐test was used to analyze. Additionally, we also found the protein of LAIR‐1 was localized in the SW‐1088 cell line. (Figure 9D).

## 4 DISCUSSION

As an immune inhibitory receptor, LAIR‐1 participates a regulatory part in immune‐related diseases and is widely expressed in hematopoietic cells. Related studies have shown that high expression of LAIR boosts the development of hematological tumor diseases such as acute myeloid leukemia^19^ and acute lymphocytic leukemia.^20^ In addition, the expression of LAIR‐1 in solid tumors such as ovarian cancer, cervical cancer, and hepatocellular carcinoma surpasses that in adjacent normal tissues and is positively correlated with tumor grade.^10^, ^21^, ^22^ These characteristics were also generally validated in our study. Given the limited literature, we conducted this study to examine the potential prognostic impact of LAIR‐1 in LGG. This is the first study to analyze the expression of LAIR‐1 in a large sample of patients with glioma.

In this study, analysis of information from the TCGA database indicated that high expression of LAIR‐1 is correlated with poor prognosis for LGG, KIRC, THYM, SKCM, and UVM. In glioma, we investigated that LAIR‐1 overexpression was interrelated with clinicopathologic features such as grade, IDH status, 1p/19q non‐co‐deletion, and MGMT, suggesting that LAIR‐1 overexpression participates a role in the malignant behavior of glioma. Both univariate and multivariate Cox regression analyses indicated that LAIR‐1 overexpression is an independent adverse factor affecting the prognosis of glioma patients, which was also confirmed in two other validated cohorts. Kaplan–Meier survival analysis was utilized to contrast the OS of patients with high and low LAIR‐1 expression, and the results showed that LAIR‐1 could be used as a prognostic indicator for LGG patients. ROC curve evaluation suggested that LAIR‐1 alone could be used to forecast the prognosis of LGG patients at 1, 3, and 5 years. However, our multifactor nomogram survival prediction model (including age, grade, IDH status, and LAIR‐1 expression) exhibited better performance than clinical features and LAIR‐1 expression alone and is therefore more beneficial for clinical treatment guidance of LGG patients.

We discovered that the expression level of LAIR‐1 in LGG patients is correlated with various immune‐related biological processes. Functional enrichment analysis found that the differential expression of LAIR‐1 in LGG patients, which has a significant impact on immune‐related biological processes, primarily manifests in neutrophil degranulation, neutrophil activation, processing and presentation of associated antigens, regulation of synaptic signaling, glycogenesis, axon genesis, regulation of neurotransmitter levels, presentation of peptide antigen via MHC, and interferon‐gama‐mediated signaling pathway. GSEA findings indicated that a high‐LAIR‐1 expression phenotype is positively correlated with processes related to leukocyte proliferation, T‐cell activation, IL‐8 production, Toll‐like receptors (TLRs), superoxide metabolic, TNF, and cytokine production. IL‐8 is a chemokine involved in maintaining the balance between physiological responses and pathological manifestations in the central nervous system.^23^ Studies have shown that IL‐8 promotes glioma invasiveness primarily by promoting angiogenesis and cell migration,^24^ which suggests that LAIR‐1 participates an critical part in mediating tumor progression via IL‐8 in the glioma TIME. Moreover, TLRs belong to the pattern recognition receptor superfamily, which typically activate and mediate the pro‐inflammatory response of innate immune cells by identifying invading pathogens.^25^ Superoxide metabolism can lead to maladjustment of cell cycle checkpoint by inducing posttranslational modification of wild‐type p53, ultimately promoting malignant tumor progression.^26^ In particular, antigen‐targeting cytotoxicity of T lymphocytes is now recognized as a critical factor in the relationship between the immune system and cancer prevention.^27^ However, cross‐linking of LAIR‐1 with its monoclonal antibody or collagen has an inhibitory effect on the activation of naïve T cells.^28^ Interestingly, our study indicates that the expression level of LAIR‐1 is closely interrelated with the above‐mentioned immune pathways, and therefore, we believe that high expression of LAIR‐1 is closely interrelated with the TIME of LGG tissues.

In essence, we identified an interrelation between tumor cells and TIME resident cells, in which the behavior of tumor cells determines the outcome of the tumor and affects the biology of TIME cells. On the contrary, resident cells of the TIME may influence tumor initiation, growth, and/or metastasis.^29^ In the latest study, the immune cells, including macrophages, micro‐glia, regulatory T cells (Tregs), myeloid‐derived suppressor cells, T lymphocytes, natural killer cells, interacts with glioma cells in the glioma..^30^, ^31^ The immune microenvironment of intracranial gliomas is heterogeneous, and the infiltrating immune cells consist primarily of microglia, peripheral macrophages, granulocytes, myeloid inhibitory cells, and T lymphocytes.^32^ Using the ESTIMATE algorithm, we discovered that stromal and immune scores in glioma patients gradually increases with the increasing expression of LAIR‐1. Moreover, heat‐map analysis also indicated that the concentration of 29 types of tumor‐infiltrating immune cells or molecules in the patients with high expression of LAIR‐1. The immune score was originally used to assess the stage and prognosis of colon cancer patients, and patients with a high immune score generally have a better prognosis.^33^ However, Kaplan–Meier's survival analysis in the present study found that the clinical outcome of patients with high LAIR‐1 expression was markedly worse than that of the control group. Therefore, we hypothesized that LAIR‐1 expression affects the type of immune infiltrating cells in LGG. TFHs aid the activity of B cells in germinal center responses and reduces immunosuppression through the inflammatory response and helps to organize tertiary lymphoid structures to achieve anti‐tumor effects, which was reported in breast, colorectal, and other tumors.^34^, ^35^ Activation and degranulation of mast cells is highly pro‐inflammatory, leading to the recruitment of cells from the immune system to coordinate the anti‐tumor immune response.^36^ Eosinophils are a subset of granulocytes, and studies have shown that IL‐33‐activated eosinophils exert immediate cytotoxic effects on tumor cells.^37^ These types of immune cells can be considered positive factors for patients with LGG. These results are in accordance with the observation in this study that LAIR‐1 expression indicative of poor prognosis is negatively correlated with the three above mentioned types of immune cells. Other types of immune‐infiltrating cells in this study had a negative impact on patient survival. For example, Treg infiltration is almost considered a marker of GBM. Other studies have shown that Treg cells inhibit the activation of effector T cells through a variety of mechanisms, and the number of Treg cells infiltrating high‐grade glioma tumors is interdependent with tumor grade and poor prognosis of in glioma.^38^, ^39^ Macrophages are divided into two types, M1 and M2. The M1 phenotype is pro‐inflammatory and anti‐tumor, whereas the M2 phenotype is cell‐protective and immunosuppressive. Notably, polarized macrophages can promote immune escape, invasion, proliferation, and angiogenesis of tumor cells.^32^, ^39^ LAIR‐1 is exactly highly enriched in macrophages, which may be involved in the immune escape and invasion of tumor cells. Resting memory CD4 cells exist in a state of cell cycle stagnation and can be activated to proliferate and rapidly differentiate only under stimulation by external specific antigens to provide auxiliary cellular immune protection.^40^, ^41^ Specifically, we considered that LAIR‐1 expression is correlated with inhibition of the activation of memory CD4 T cells and mast cells, inhibition of the aggregation of TFHs and eosinophils, and promotion of the polarization of M2 macrophages and aggregation of Treg cells. However, the specific mechanism requires further study.

The development of new immunotherapies has advanced rapidly in the field of oncology in recent years, and immune checkpoint inhibition is considered a potentially important method for the treatment of adult glioma. Mechanisms that suppress the activation and/or effector function of immune cells are called immune checkpoints.^42^, ^43^ In this study, we examined the dependence between LAIR‐1 expression and various immune checkpoints. Our result showed that LAIR‐1 expression is strongly interrelated with CD86 and moderately with CD80, meaning that the physiological mechanisms of LAIR‐1 and CD86 are highly similar. CD86 was shown to be a poor prognostic factor in LGG, SKCM, and UVM, similar to our findings in the pan‐cancer analysis.^44^ CD86 (B7‐2), CD276 (B7‐H3), and CD80 (B7‐1) are immunoglobulin‐like proteins expressed by antigen‐presenting cells and interact with CD28 and cytotoxic T lymphocyte‐associated antigen‐4 (CTLA‐4) receptors on T cells. Their interactions with CD28 serve a costimulatory function to promote T cell proliferation and antigen responses by activated T cells, but their interactions with CTLA‐4 lead to produce immunosuppression of effector T cell responses.^45^, ^46^, ^47^ Our results indicated that the expression of LAIR‐1 is poorly correlated with CD28, which suggests that LAIR‐1 is highly involved in immunosuppression of the B7 (especially CD86) and CTLA‐4 axes. In addition, LAIR‐1 includes two ITIMs in its intracellular segment, and the ITIM receptors are an ideal target for tumor immunotherapy. Indeed, considerable progress has been made in targeting these receptors, such as PDCD1/PD‐L1(also known as CD274), CD47 blockade therapies.^48^, ^49^ A recent tumor study in mice found that LAIR‐2‐Fc recombinant protein inhibits the binding of LAIR‐1 and collagen by blocking the PD‐1/PD‐L1 checkpoint, so as to achieve an anti‐tumor effect.^50^ These studies show that LAIR‐1 participates a critical part in the PD‐1/PD‐L1 axis, similar to our results in that LAIR‐1 expression was positively correlated with PD‐1 and PD‐L1. It is worth noting that the first generation of immune checkpoint inhibitors, such as inhibitors of CTLA‐4 and PD‐1, targeted the most distinctive immune checkpoints and thus represent the most mature immunotherapy agents.^42^, ^51^ Moreover, LAIR‐1 expression was moderately correlated with other second‐generation immune checkpoint genes, including CD40, TNFSF14, BTLA, and TNFRSF14. Thus, we considered the possibility of LAIR‐1 as a new therapeutic target for LGG patients, as it could provide an important new basis and direction for immunotherapy in glioma patients. As tumor immune resistance is characterized by the co‐expression of multiple immune checkpoint pathway molecules, double or multiple checkpoints blocked may produce more powerful anti‐tumor immunotherapy effects; therefore, additional immune targets need to be identified.

There are many restrictions in the present study. First, this study was based on preliminary data and hypothesis‐generating predictions. Second, although we found that LAIR‐1 expression was interrelated with patient prognosis and immune invasion in glioma, we could not prove that LAIR‐1 affects prognosis through immune invasion, which needs to be verified using different cell lines. In addition, elucidating the mechanisms by which LAIR‐1 regulates the infiltration of immune cells will require further study. The experimental evidence of whether the expression of LAIR‐1 will affect the abundance of immune cell infiltration is a deficiency of our study, and it will become a direction of follow‐up research. However, this subject is new and worthy of further study.

In conclusion, our study suggests that LAIR‐1 is a latent marker for determining the prognosis of LGG patients. LAIR‐1 may also participate a critical part in TIME of LGG by regulating the infiltration of immune cells, suggesting that LAIR‐1 might be used as a therapeutic target to regulate the anti‐tumor immune response.

## AUTHOR CONTRIBUTIONS

Zhansheng Fang, Li Linand Zewei Tumainly responsible for writing the manuscript and figure production. Lei Wu, Xingen Zhuand Kai Huangsupervise the work. Pengxiang Luoand Jingying Liare devoted to data collecting. All authors consent the results and contribute to the final manuscript.

## FUNDING INFORMATION

The current study was funding by Natural Science Foundation of Jiangxi Province (grant nos. 20192BAB205077), the National Natural Science Foundation (grant nos. 81860448 and 82002660), and Jiangxi Province Department of Education Science and Technology Research project, China (grant no. GJJ190018).

## CONFLICT OF INTEREST

The authors declare no potential conflicts of interest.

## ETHICS STATEMENT

The ethics committee of the Second Affiliated Hospital of Nanchang University reviewed and approved the study involving human participants

## Supporting information


Figure S1

Figure S2

Figure S3

Figure S4

Figure S5

Figure S6

## Data Availability

The datasets presented in this study can be found in online database. The names of the database and accession number(s) can be found in the article/Supplementary Material.
