# Supplementary figures and images for: Development and validation of a leukocyte‐associated immunoglobulin‐like receptor‐1 prognostic signature for lower‐grade gliomas
Source: Cancer Med. 2022 Jun 15;12(1):712–32. doi: 10.1002/cam4.4945 (PMC9844621; doi:10.1002/cam4.4945)

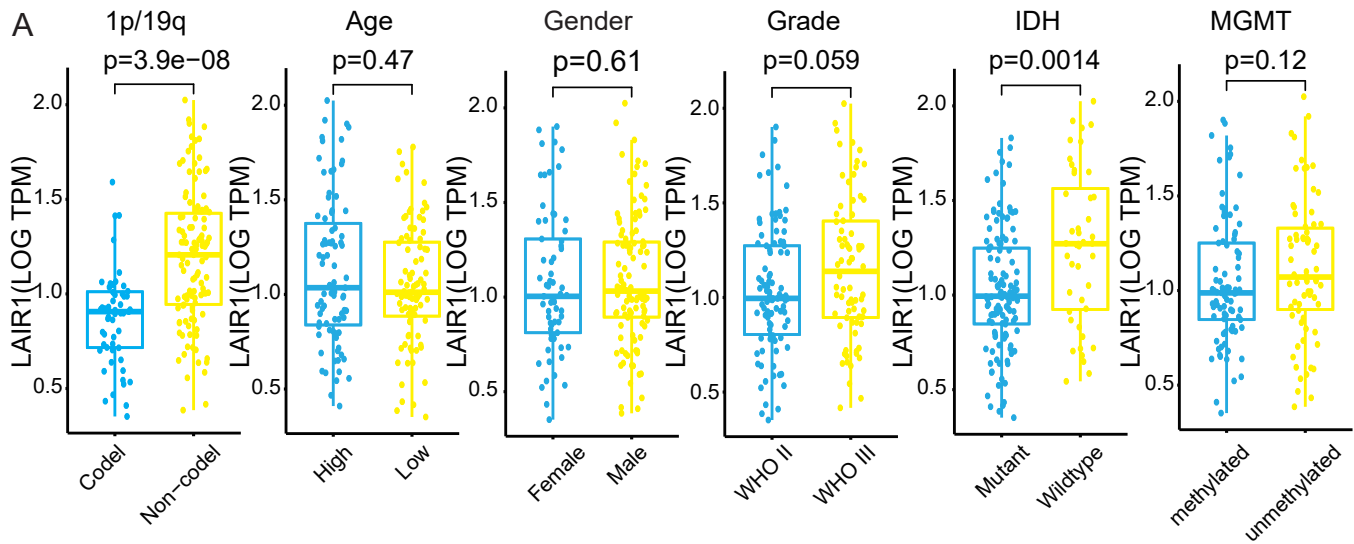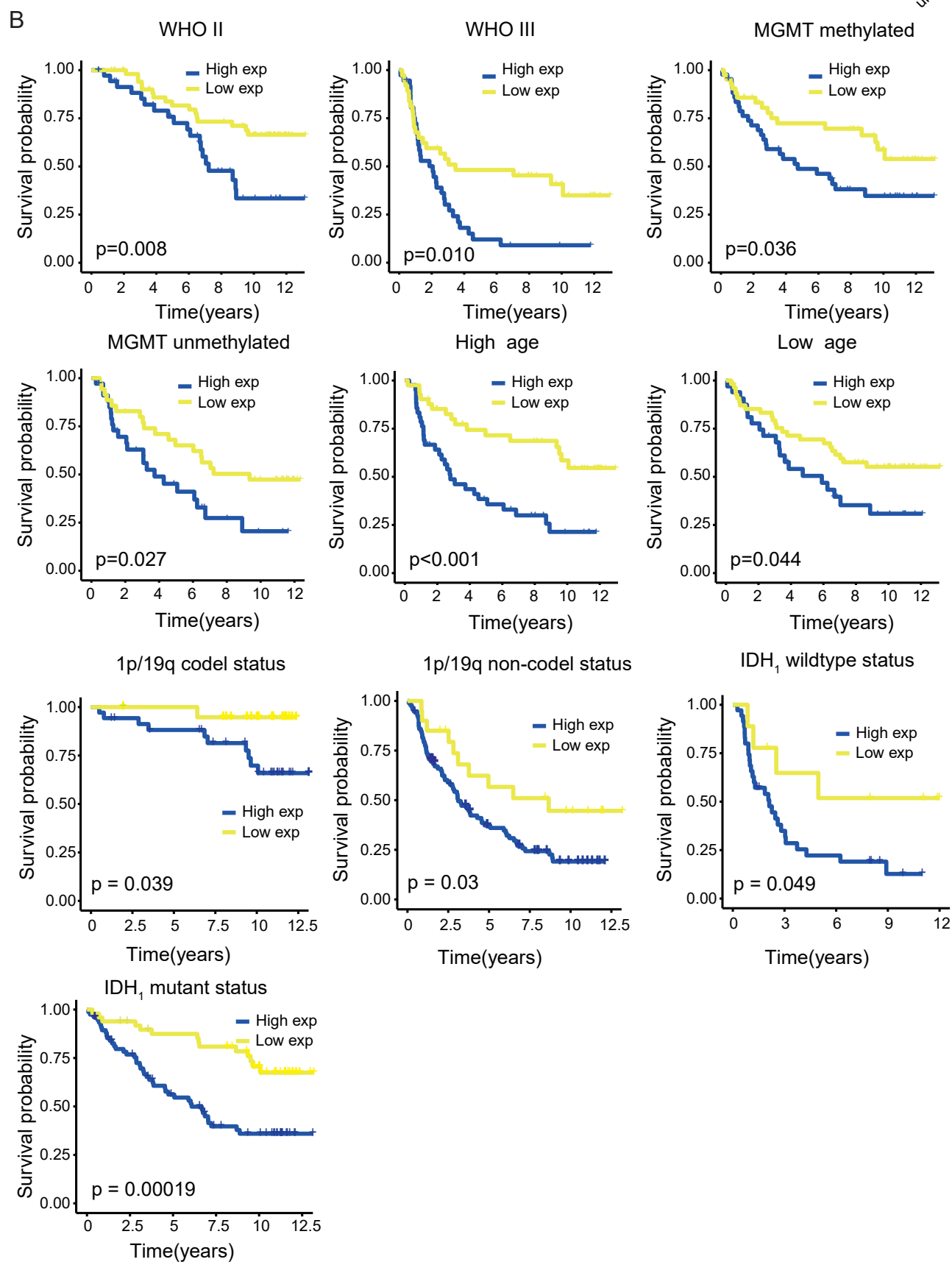

Supplement: Supplementary file 1 — Figure S1 [file CAM4-12-712-s005.pdf]

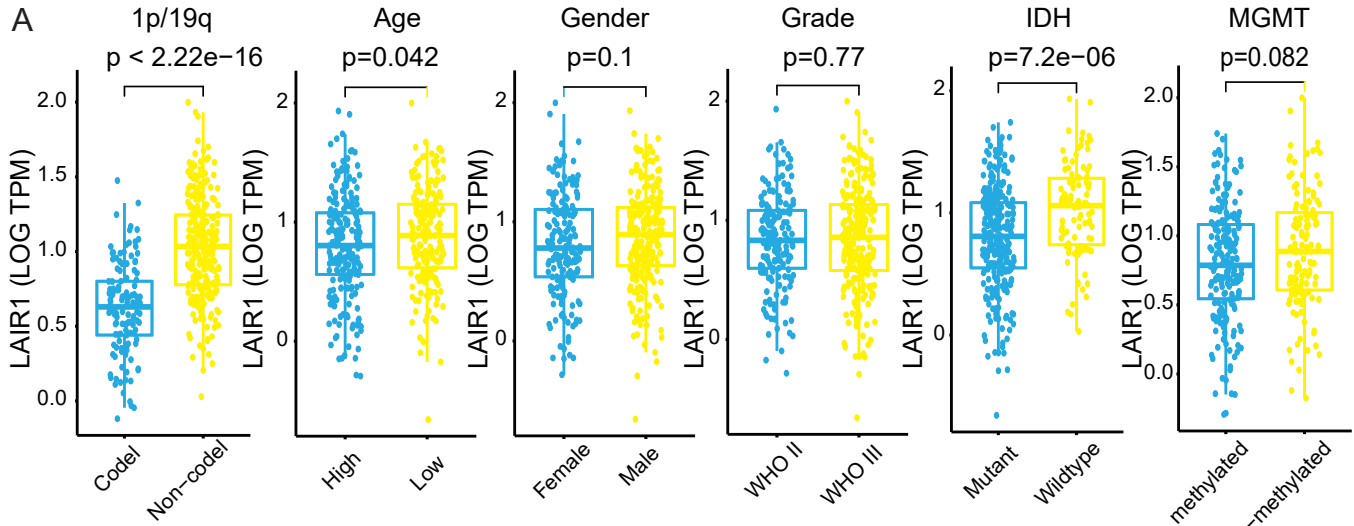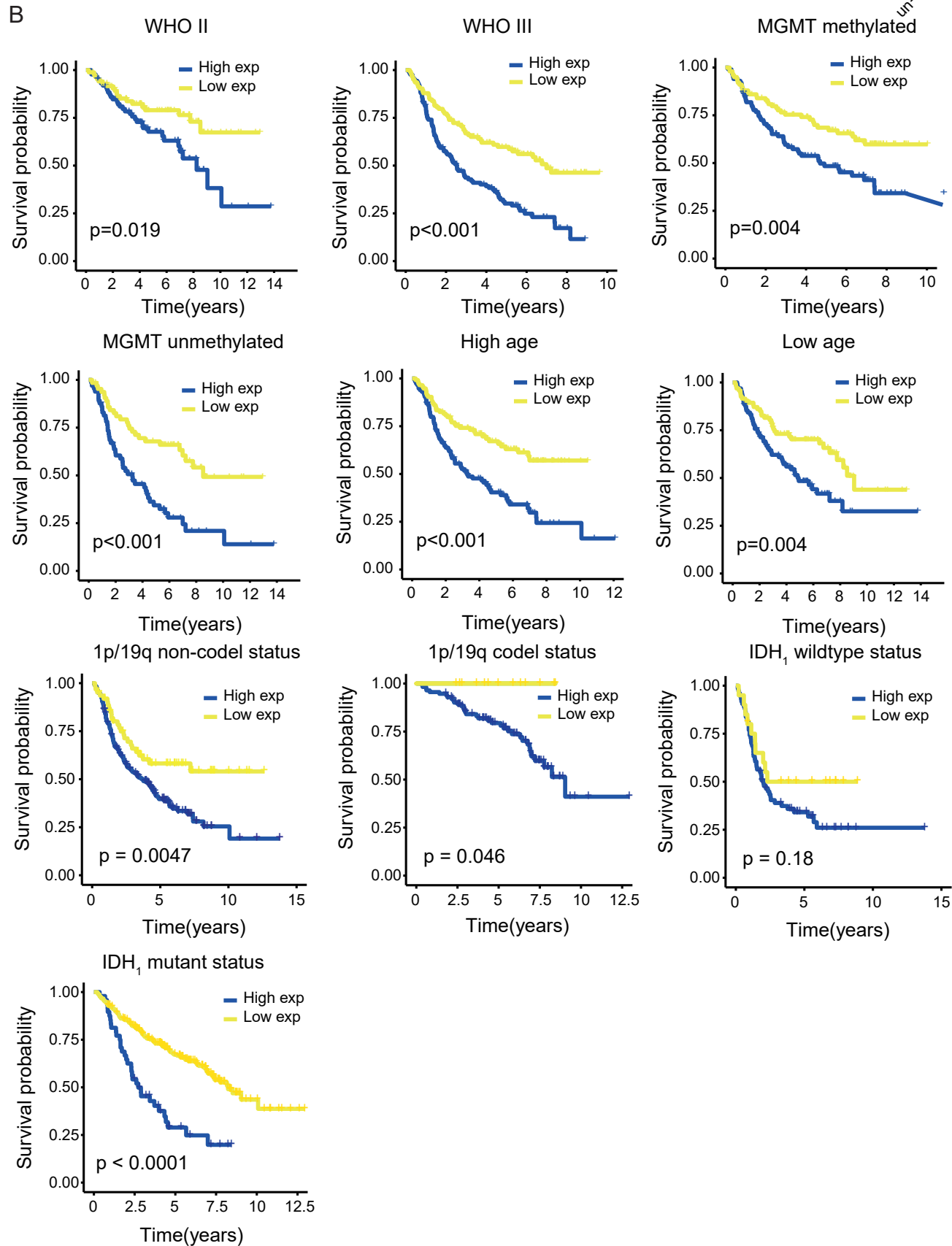

Supplement: Supplementary file 2 — Figure S2 [file CAM4-12-712-s001.pdf]

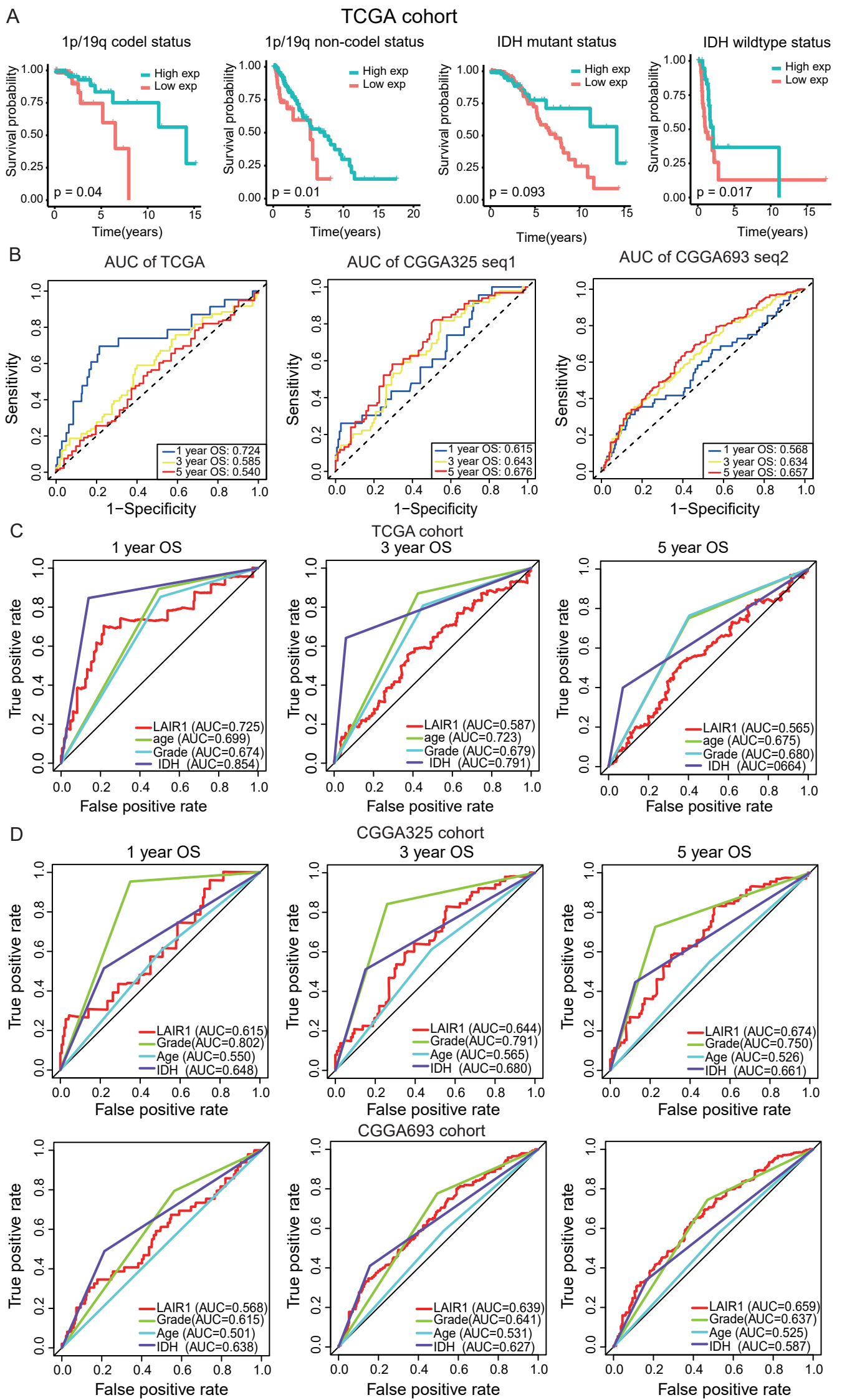

Supplement: Supplementary file 3 — Figure S3 [file CAM4-12-712-s004.pdf]

## GSE16011 cohort

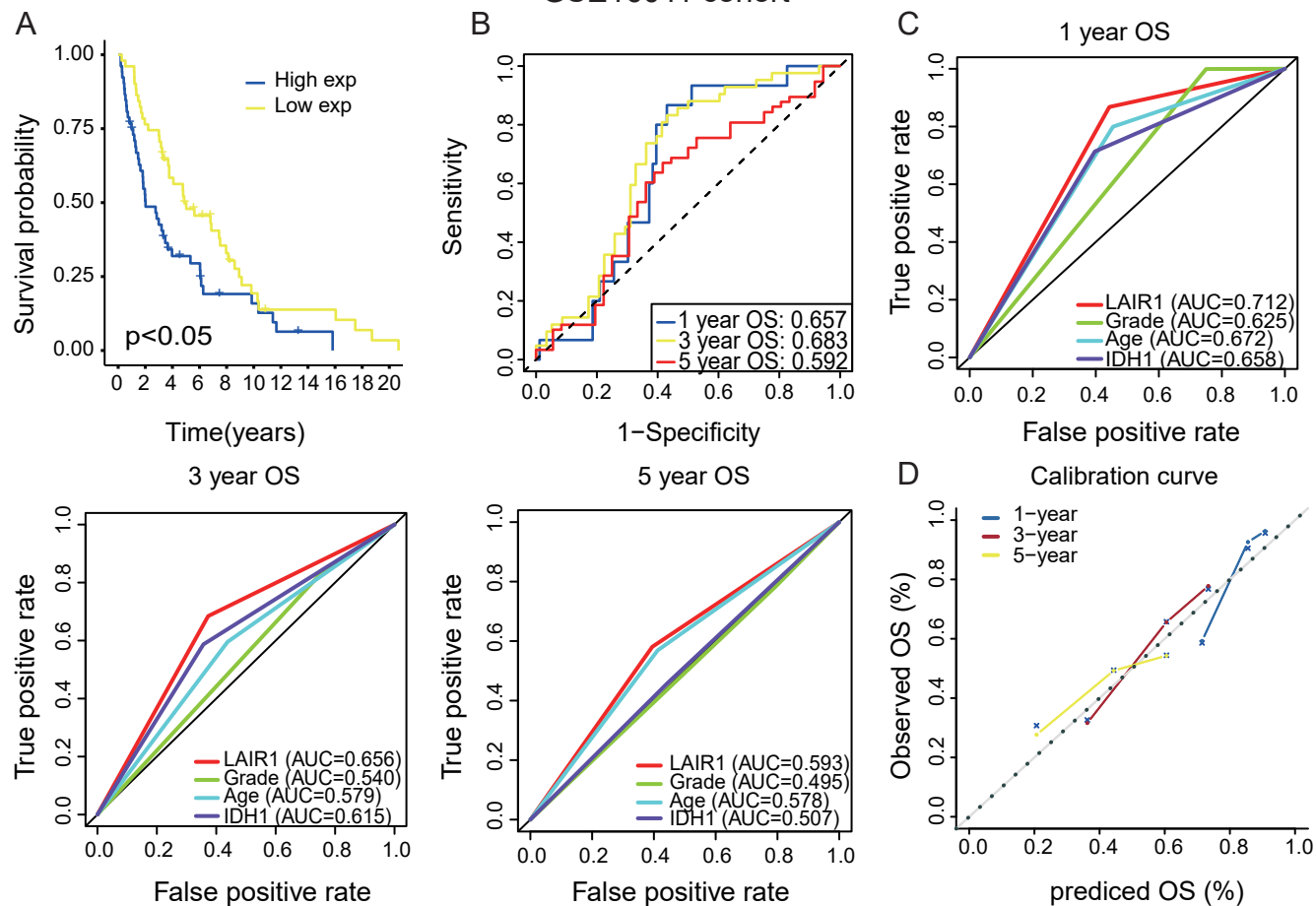

## GSE61374 cohort

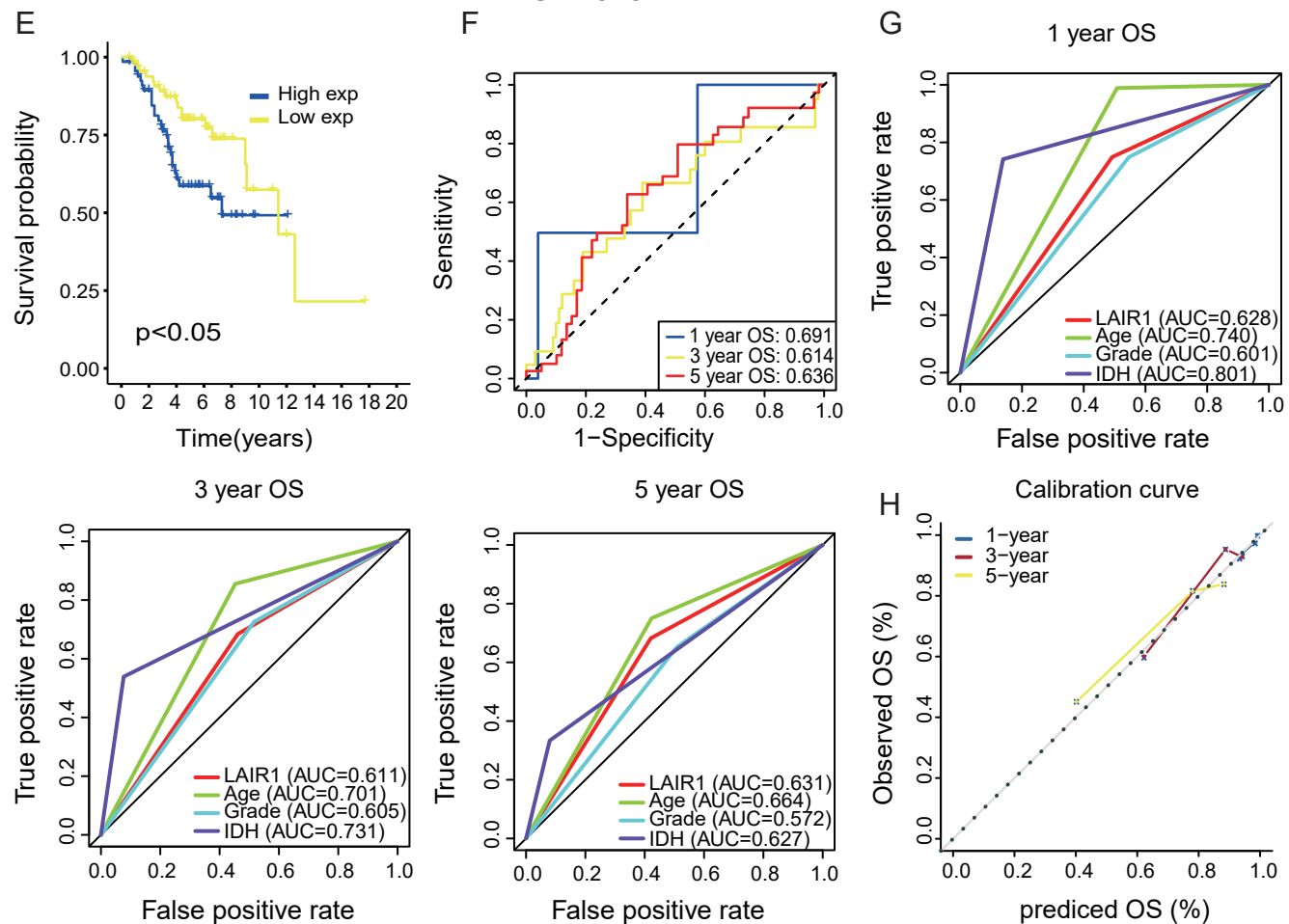

Supplement: Supplementary file 4 — Figure S4 [file CAM4-12-712-s002.pdf]

A

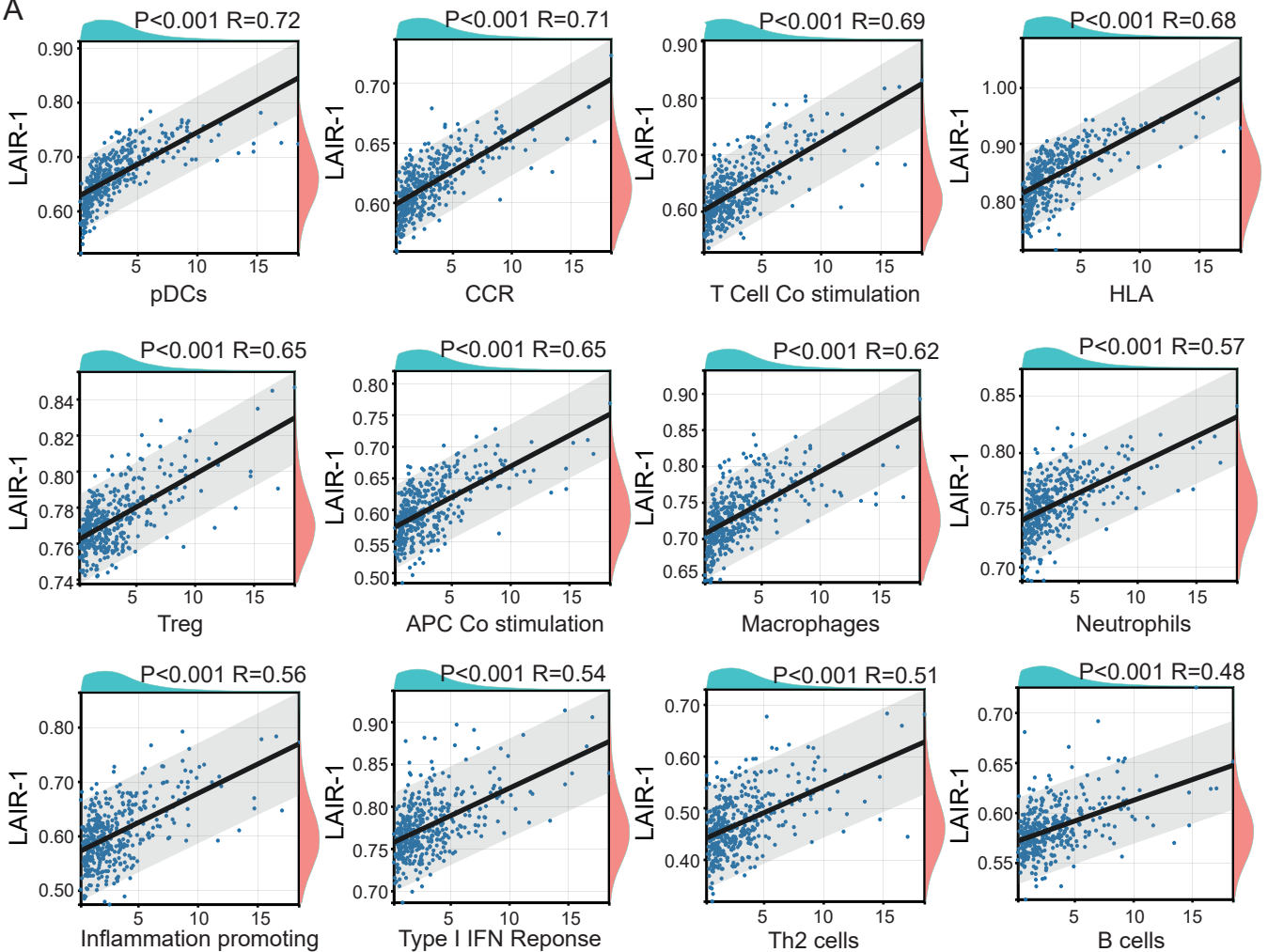

Supplement: Supplementary file 5 — Figure S5 [file CAM4-12-712-s006.pdf]

A

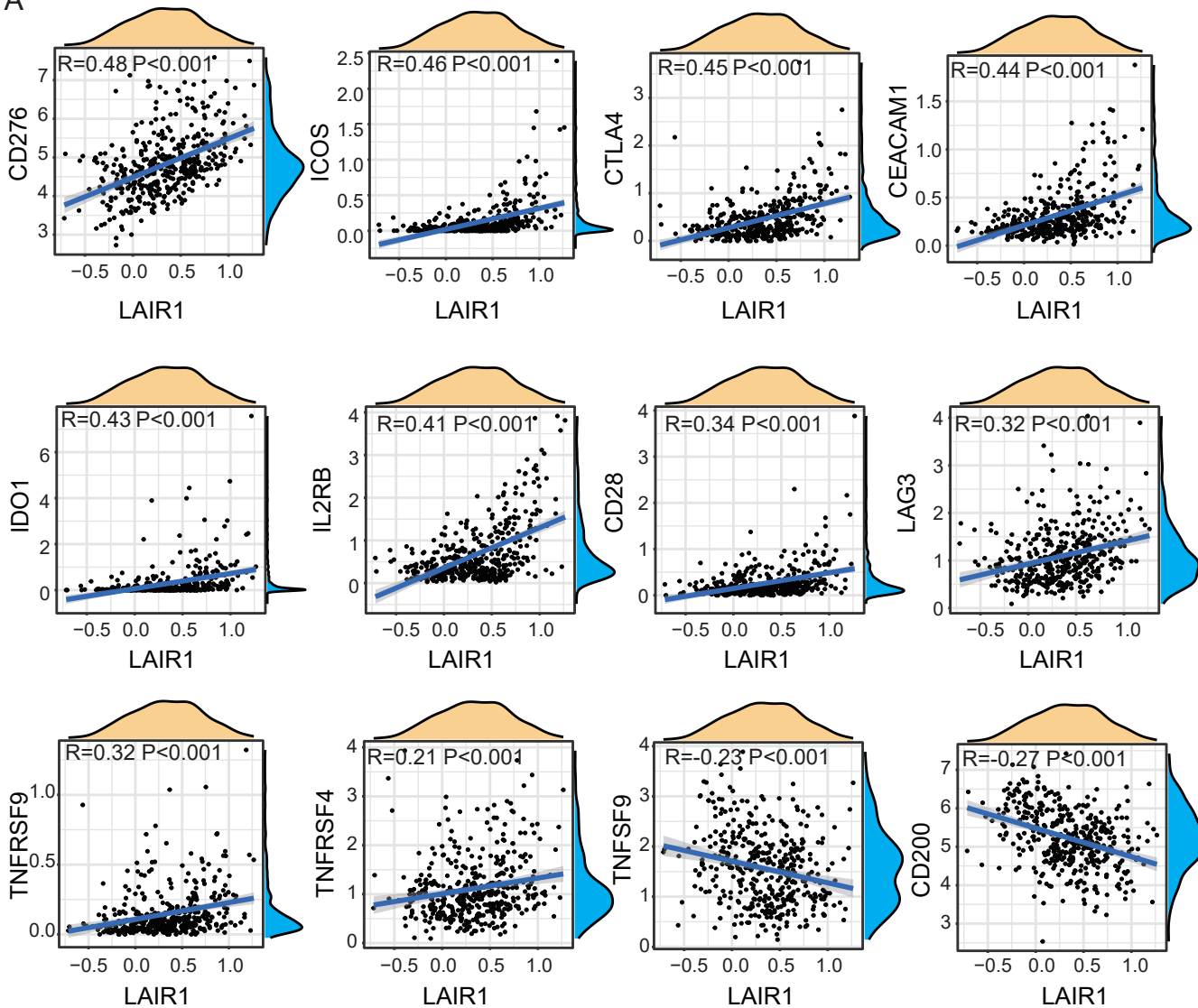

B

### IHC staining of LAIR1 in gliomas

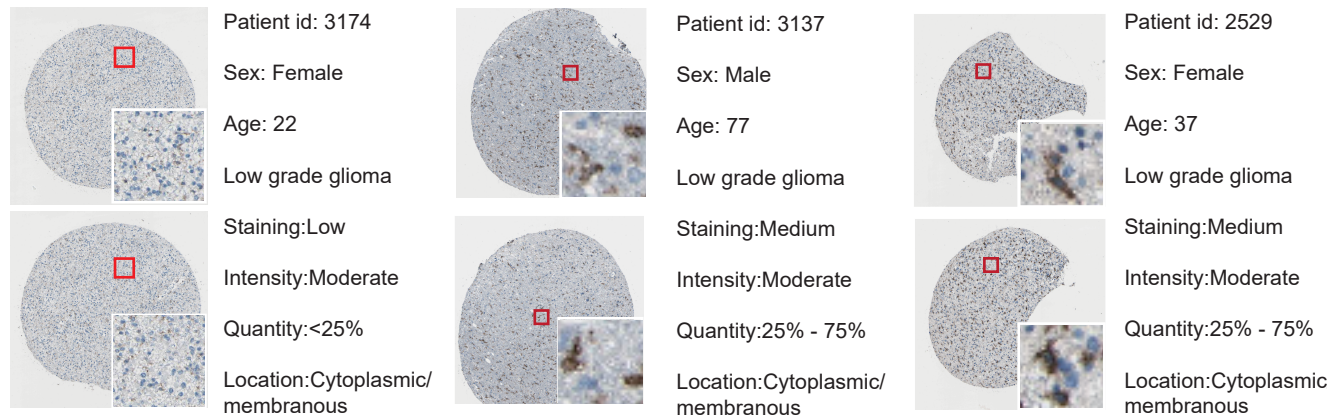

Supplement: Supplementary file 6 — Figure S6 [file CAM4-12-712-s003.pdf]
